# Prevalence and Long-Term Prognostic Significance of Advanced Diastolic Dysfunction Among Hospitalized Patients Referred for Echocardiography

**DOI:** 10.3390/jcm14041096

**Published:** 2025-02-08

**Authors:** Ziv Dadon, Mady Moriel, Abdallah Tirhi, Amjad Abu Salman, Michael Glikson, Shemy Carasso, Shmuel Gottlieb

**Affiliations:** 1 Jesselson Integrated Heart Center, Eisenberg R&D Authority, Shaare Zedek Medical Center, Jerusalem 9103102, Israel; ziv.dadon@mail.huji.ac.il (Z.D.);; 2 Faculty of Medicine, Hebrew University of Jerusalem, Jerusalem 9112102, Israel; 3 Azrieli Faculty of Medicine, Bar-Ilan University, Zefat 1311502, Israel; 4 Sackler Faculty of Medicine, Tel Aviv University, Tel Aviv 6997801, Israel

**Keywords:** diastolic dysfunction, predictors, prognosis, five-year mortality, outcome assessment, admission

## Abstract

**Background/Objectives:** Left ventricular diastolic dysfunction (LVDD) is associated with unfavorable outcomes, and though it is recognized as an important clinical diagnosis, specific quantification and effective management continue to challenge clinicians, representing an unmet need in modern cardiology. Advanced LVDD diagnosis is likely to have a prognostic role among hospitalized patients. The aim of this study was to describe the prevalence and predictors of advanced LVDD among hospitalized patients and its long-term (5-year) prognostic significance on all-cause mortality. **Methods:** This was a retrospective observational study of consecutive, non-selected hospitalized patients referred for echocardiography at a tertiary care medical center from October 2013 to February 2024. Diastolic function was classified into normal/LVDD grade I vs. advanced LVDD (grades II and III). **Results:** A total of 5926 participants were included in the analysis, of whom 3229 (54%) were men, with a mean age of 66 ± 2 years. These included 4779 (81%) patients with normal/LVDD grade I and 1147 (19%) with advanced LVDD. Compared to patients with normal/LVDD grade I, those with advanced LVDD were older, were more likely to be men, and had a higher burden of hypertension, diabetes mellitus, congestive heart failure, atrial fibrillation/flutter and renal failure, abnormal laboratory findings, worse echocardiogram parameters, and longer hospital stay. Multivariate analysis revealed that advanced LVDD was independently associated with increasing age, the male sex, significant aortic stenosis, hypertension, and atrial fibrillation. Patients with advanced LVDD vs. normal/LVDD grade I had higher 5-year all-cause mortality rates (p_log-rank_ < 0.001). Multivariate Cox proportional hazards regression model revealed that advanced LVDD was associated with a 24% increase in the 5-year mortality rate (HR = 1.236, 95% CI of 1.008–1.517, *p* = 0.042). **Conclusions:** Among hospitalized patients referred for echocardiography, the prevalence of advanced LVDD was 19%, and it was independently associated with age, the male sex and the presence of multiple comorbidities. Moreover, advanced LVDD was identified as an independent predictor of long-term all-cause mortality. Advanced LVDD should be proactively diagnosed among admitted patients at risk for early therapy tailoring.

## 1. Introduction

Left ventricular diastolic dysfunction (LVDD) has been associated with unfavorable outcomes including increased cardiovascular death, atrial fibrillation (AF), and hospitalization for heart failure. However, the pathophysiologic mechanisms linking LVDD to clinical events have been poorly identified [1,2]. Risk factors for LVDD include aging, sex, hypertension, lipid disorders, type 2 diabetes mellitus, baseline structural heart disease, and metabolic syndrome, though the exact mechanism of some of these factors is not thoroughly understood [3,4,5,6]. Though it is recognized as an important clinical diagnosis, the specific quantification and effective management of LVDD continue to challenge clinicians and represent a growing unmet need in modern cardiology.

The primary modality used for diagnosing and categorizing LVDD in routine clinical practice is transthoracic echocardiography (TTE). Different protocols have been suggested for the diagnosis of LVDD [7,8]. The hallmark of these approaches, which has been emphasized by the American Society of Echocardiography (ASE) and the European Association of Cardiovascular Imaging (EACVI) recommendations [8], is the initial differentiation between preserved and reduced LV systolic function followed by the evaluation of four critical TTE-based indices, with subsequent calculation of a three-grade LVDD severity scale.

Based on these recommendations, previous studies have been published describing LVDD prevalence and outcome [9,10]. However, they were mostly conducted on the general population and were not specific to selected patient subgroups, e.g., hospitalized individuals. As such, there is a dearth of data regarding the role of LVDD among hospitalized patients, a highly vulnerable group with limited physiological reserves and a higher rate of unfavorable outcomes. This study aimed to describe the prevalence of advanced LVDD among hospitalized patients and to assess its predictors and prognostic significance focusing on long-term all-cause mortality.

## 2. Materials and Methods

### 2.1. Study Setting

This is a retrospective observational study including consecutive, non-selected hospitalized patients referred for an echocardiogram at a tertiary care medical center from 28 October 2013 to 12 February 2024. This study was approved by the Institutional Review Board (IRB; 028522-SZMC) with a waiver of informed consent.

### 2.2. Study Endpoints

The aims of this study were to describe the prevalence of advanced LVDD among hospitalized patients who underwent TTE, as well as to evaluate variables associated with this finding and its association with long-term (5-year) all-cause mortality.

### 2.3. LV Diastolic Function Assessment

LVEF was assessed based on apical views (using visual assessment or the modified Simpson’s method). LA area was measured using planimetry on the end-systolic apical four-chamber (A4ch) view and LA volume by using the modified Simpson’s method in the end-systolic A4ch and apical two-chamber views. This value was then indexed to the body surface area (BSA). Trans-mitral inflow velocities were assessed at the leading edge of the spectral waveform using the pulsed-wave Doppler (apical four-chamber view) for peak early (E) and peak late (A) mitral inflow velocities (cm/sec). E/e′ was calculated as early mitral inflow velocity divided by the average of septal and lateral mitral annular peak early diastolic velocity (e′) obtained by pulsed-wave tissue Doppler imaging (TDI). Diastolic function was assessed among patients with normal/preserved LV systolic function (LVEF ≥ 50%) based on the ASE/EACVI recommendations [8]. The following variables were evaluated with their corresponding abnormal cutoff values:

(1) Septal e′ < 7 cm/sec or lateral e′ < 10 cm/sec; (2) average E/e′ ratio > 14; (3) peak TR velocity > 2.8 m/sec; (4) LA volume index > 34 mL/m^2^.

LVDD was diagnosed when at least 2 of these 4 variables met the cutoff values.

### 2.4. LVDD Classification

Once diagnosed, LVDD’s severity was categorized based on the following criteria:

(A) Grade 0: normal diastolic function; (B) Grade I: E/A < 0.8 and E ≤ 50 cm/s; (C) Grade II: E/A < 0.8 and E > 50 cm/s or 0.8 < E/A < 2 cm/s (only in case that at least 2 of the above-mentioned 2–4 variables met their relevant cutoff thresholds); (D) Grade III: E/A > 2 cm/s. Classification into either of these categories was not based on documented rhythm (sinus rhythm, paced rhythm, or other).

### 2.5. Study Protocol

All echocardiographic exams were acquired by a certified echocardiographic technician (equivalent to a Registered Diagnostic Cardiac Sonographer) using a high-end echocardiography device. The clips were then evaluated by an echocardiographic cardiologist for relevant echocardiographic indices. Significant aortic stenosis (AS) was defined as a moderate-severe/severe degree. The study excluded patients younger than 18 years, those with reduced LVEF (<50%), mitral stenosis, AF rhythm during the exam, and those with insufficient Doppler data to enable diastolic function assessment. Repeated echocardiograms during the index admission were excluded as well.

Data of the study participants were analyzed for demographics, baseline characteristics, medical history, admission course, blood workup, and echocardiographic parameters as demonstrated by the index echocardiogram. Medical history, including AF, was based on the records provided by the treating physician’s assessment. These variables were collected based on the hospital’s computerized database. The patients were followed until the time of death or up to 5 years, whichever came first. All-cause mortality data were derived and confirmed by the National Israeli Population Registry of the Ministry of Interior.

### 2.6. Statistical Analyses

Study participants were divided into two groups based on their LVDD severity classification: normal or LVDD grade I vs. advanced LVDD (grade II and III). Analyses were performed accordingly. Patients’ characteristics were described by mean ± SD for continuous variables and numbers with percentages for categorical variables. Descriptive statistics were used to analyze differences in baseline and clinical characteristics, echocardiogram parameters, admission course, and outcomes using chi-square tests for categorical variables and the unpaired Student *t*-test or Mann–Whitney U test for continuous variables comparison. Test selection was based on data distribution and normalcy.

Unadjusted odds ratios (ORs) with 95% confidence intervals (CIs) were calculated to test the associations between advanced LVDD and the study variables, including patient baseline characteristics and medical history. Multivariate logistic regression analysis (OR with 95% CI) was conducted in order to identify variables independently associated with advanced LVDD (*p* ≤ 0.05), using the enter method. Kaplan–Meier cumulative survival analysis was conducted to compare all-cause mortality of patients with advanced LVDD vs. normal/LVDD grade 1. The differences between the two groups were assessed using the log-rank test. A univariate Cox proportional hazard was calculated for each of the pertinent variables to assess the HR with 95% CI for time to all-cause mortality or up to 5 years, whichever came first. Multivariate Cox proportional hazards regression model with HR and 95% CI was used to calculate time to all-cause mortality including variables that were found significant (*p* ≤ 0.05) in the univariate analysis and included the following variables: advanced LVDD, age, male sex, obesity, renal failure, stroke/TIA, AF/AFL, diabetes mellitus, hypertension, hyperlipidemia, IHD, chronic obstructive pulmonary disease (COPD), coronary artery bypass grafting (CABG), and cardiology ward admission. To account for potential year- and time-related biases, the year of admission was included in the multivariate analysis as a covariate.

Statistical analyses were performed using the SPSS Statistics for Windows version 21 (SPSS Inc., Chicago, IL, USA). All tests were two-sided with a *p*-value ≤ 0.05 considered statistically significant.

## 3. Results

A total of 5926 participants were included in the analysis, of whom 3229 (54%) were men, with a mean age of 66.0 ± 20.5 years; 2385 (40%) were >75 years (Table 1). These included 4779 (81%) patients with normal/LVDD grade I and 1147 (19%) with advanced LVDD (790 [13%] with LVDD grade II and 357 [6%] with LVDD grade III).

### 3.1. Baseline Characteristics and Comorbidities of Patients with Normal/LVDD Grade I vs. Advanced LVDD

As detailed in Table 1, compared to patients with normal/LVDD grade I, those with advanced LVDD were older (72.5 ± 21.2 vs. 64.5 ± 20.1 years, *p* < 0.001), more likely to be male (64% vs. 52% *p* < 0.001), and more likely to have hypertension, diabetes mellitus, congestive heart failure (CHF), AF/atrial flutter (AFL), and renal failure, but fewer were smokers. The two subgroups had similar rates of obesity, hyperlipidemia, ischemic heart disease (IHD), coronary interventions, stroke/TIA, and COPD. Similar results were obtained for patients who met the primary outcome or completed the 5-year follow-up (Supplementary Table S1).

### 3.2. Admission Characteristics of Normal/LVDD Grade I vs. Advanced LVDD

As detailed in Table 1, compared to patients with normal/LVDD grade I, those with advanced LVDD had higher rates of intravenous furosemide treatment (34% vs. 30%, *p* < 0.001) and a longer length of stay (median of 9.1 [5.5–16.8] vs. 8.9 [4.6–17.0] days, *p* = 0.029). The highest proportion of patients with advanced LVDD were admitted to the cardiology ward, followed by the internal medicine and surgery wards (23.9% vs. 20.0%, 16.2%, *p* < 0.001). Individuals with advanced LVDD had higher levels of NT-pro-BNP, creatinine, and potassium, but lower levels of hemoglobin. The groups did not differ in peak troponin-T and TSH levels.

### 3.3. Echocardiogram Characteristics of Normal/LVDD Grade I vs. Advanced LVDD (Table 2)

As detailed in Table 2, compared to patients with normal/LVDD grade I, those with LVDD grade II/III had higher rates of significant AS, wider interventricular septum, shorter LV internal diastolic diameter, longer LA short axis diameter, larger LA volume and LA volume index, greater E wave velocity, A wave velocity, E/A ratio, E/e′ ratio, and tricuspid insufficiency gradient with lower e′ septum and e′ lateral velocities. The groups did not differ in the LA area or LV internal systolic diameter. Similar results were obtained for patients who met the primary outcome or completed the 5-year follow-up (Supplementary Table S2).

**Table 2:** Echocardiographic parameters of patients with normal/LVDD grade I vs. advanced LVDD (grade II and III).

Variable	All Patients *n* = 5926	Normal/LVDD Grade I *n* = 4779	LVDD Grade II/III *n* = 1147	*p*-Value
HR, bpm (mean ± SD)	76.2 ± 20.8	76.9 ± 21.9	73.5 ± 15.5	<0.001
Significant AS, % *	4.3	3.4	7.9	<0.001
**Ventricular characteristics**				
LVDD, cm (mean ± SD)	4.6 ± 1.4	4.6 ± 1.6	4.5 ± 0.6	0.016
LVSD, cm (mean ± SD)	2.9 ± 0.5	2.9 ± 0.5	2.8 ± 0.6	0.058
LVPWD, cm (mean ± SD)	1.0 ± 0.2	1.0 ± 0.2	1.0 ± 0.2	<0.001
IVSD, cm (mean ± SD)	1.2 ± 0.3	1.2 ± 0.4	1.3 ± 0.3	<0.001
LVEF, % (mean ± SD)	57.9 ± 4.1	58.0 ± 4.0	57.5 ± 4.3	0.002
**Atrial characteristics**				
LA diameter, cm (mean ± SD)	3.9 ± 0.8	3.9 ± 0.6	4.1 ± 1.2	<0.001
LA area, cm^2^ (mean ± SD)	24.1 ± 11.4	22.9 ± 8.5	26.0 ± 14.8	0.178
LA volume, cm^3^ (mean ± SD)	68.0 ± 40.8	64.4 ± 37.0	76.6 ± 47.7	<0.001
LA volume index, mL/m^2^ (mean ± SD)	37.2 ± 25.7	34.6 ± 19.9	43.2 ± 35.1	<0.001
LA volume index > 34 mL/m^2^, %	48.6	38.8	71.6	<0.001
**Waves**				
E wave velocity, cm/s (mean ± SD)	83.8 ± 69.4	81.3 ± 26.2	94.2 ± 148.0	<0.001
A wave velocity, cm/s (mean ± SD)	84.4 ± 33.7	82.7 ± 32.2	91.4 ± 38.6	<0.001
e′ septum velocity, cm/s (mean ± SD)	7.3 ± 2.9	7.4 ± 2.9	6.7 ± 2.8	<0.001
e′ lateral velocity, cm/s (mean ± SD)	9.3 ± 3.7	9.5 ± 3.6	8.5 ± 4.1	<0.001
**Waves ratio**				
E/A ratio (mean ± SD)	1.1 ± 1.9	1.0 ± 0.4	1.5 ± 4.2	<0.001
E/A ratio > 2, %	6.0	0	31.1	<0.001
E/e′ ratio (mean ± SD)	11.1 ± 5.5	10.6 ± 5.3	13.1 ± 5.9	<0.001
E/e′ ratio > 14, %	19.8	16.3	34.8	<0.001
**Gradient**				
TIG, mmHg (mean ± SD)	34.0 ± 15.2	32.3 ± 14.8	40.2 ± 15.1	<0.001
TIG > 28 mmHg, %	56.8	50.0	81.0	<0.001

* Defined as moderate-severe or severe aortic stenosis. Abbreviations. bpm, beat per minute; cm, centimeter; HR, heart rate; IVSD, interventricular septal diameter; LA, left atrium, LVDD, left ventricular diastolic dysfunction; LVEF, left ventricular ejection fraction, LVDD, left ventricular diastolic diameter; LVSD, left ventricular systolic diameter; LVOT, left ventricular outflow tract; LVPWD, left ventricular posterior wall diameter; m, meter; ml, millimeter; mmHg, millimeter of mercury; SA, short axis; SD, standard deviation; TIG, tricuspid insufficiency gradient.

### 3.4. Unadjusted and Adjusted Variables Associate with Advanced LVDD

The unadjusted associations between pertinent variables and advanced LVDD are presented in Table 3 and Supplementary Figure S1. Multivariate analysis revealed that advanced LVDD was independently associated with increasing age, male sex, significant AS, hypertension, and AF/AFL (Table 3 and Supplementary Figure S2).

### 3.5. Outcome

#### 3.5.1. Unadjusted Mortality

**Figure 1:**
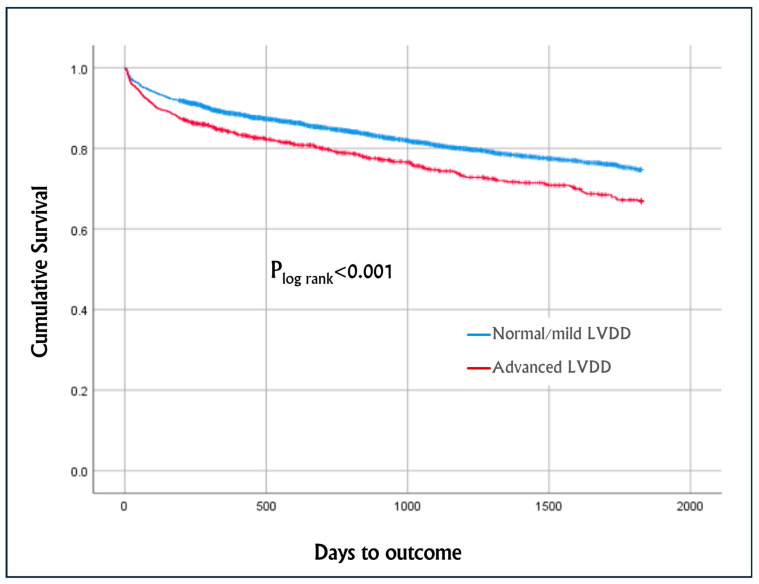
Five-year Kaplan–Meier cumulative survival curves comparing normal/mild left ventricular diastolic dysfunction (LVDD) vs. advanced LVDD (grade II/III). The differences between the groups were assessed by the log-rank test.

Figure 1 describes the Kaplan–Meier cumulative survival curves of the entire cohort, which was worse among patients with advanced LVDD vs. normal/mild LVDD, with a mean survival of 1422 days (95% CI 1382–1463) and 1525 days (95% CI 1508–1543), respectively (p_log-rank_ < 0.001).

The prevalence of 5-year mortality and survival of various pertinent demographics and comorbidities are presented in Supplementary Table S3 and Figure S3. The unadjusted HRs with 95% CI of each of the pertinent variables associated with mortality are presented in Supplementary Table S3 and Figure S4. Five-year mortality was higher among patients with hypertension, diabetes mellitus, renal failure, advanced age, hyperlipidemia, AF/AFL, IHD, COPD, and among patients with advanced LVDD (OR_unadj_ = 1.392 [1.221–1.586], *p* < 0.001). In contrast, mortality was lower among patients with stroke/TIA, obesity, post-CABG and those admitted to the cardiology ward.

#### 3.5.2. Adjusted Mortality

**Figure 2:**
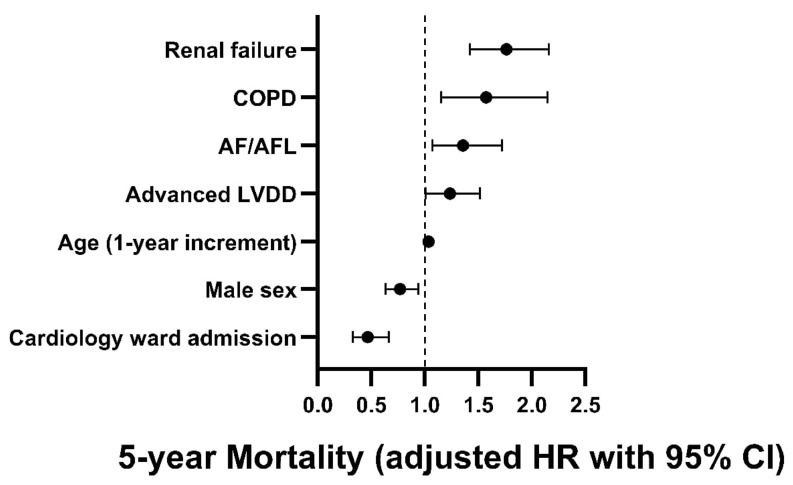
Variables independently associated with 5-year all-cause mortality *. * Multivariate Cox proportional-hazards regression analysis using hazard ratio with 95% confidence interval^.^ included the following variables: sex and variables found significant (*p* ≤ 0.05) in the univariate HR analysis. Other variables included in the model that did not show significance included year of admission, DM, hypertension, hyperlipidemia, ischemic heart disease, obesity, stroke/TIA, and CABG (see Supplementary Table S4 for full details). Abbreviations as prior Tables.

As detailed in Figure 2 and Supplementary Table S4, the Cox proportional-hazards regression analysis adjusting for pertinent variables (see Section 2: Materials and Methods) revealed that advanced LVDD was independently associated with a 24% increase in the rates of 5-year mortality (HR = 1.236, 95% CI of 1.008–1.517, *p* = 0.042). Other variables that were independently associated with increased 5-year mortality included increasing age, renal failure, AF/AFL, and COPD, whereas male sex, and admission to the cardiology ward were associated with a better outcome. These variables were found to be significantly associated with increased mortality even after omitting advanced LVDD from the model.

## 4. Discussion

The present study assessed the prevalence and long-term, up to 5 years, all-cause mortality of advanced LVDD among hospitalized patients in a tertiary medical center in the years 2013–2024 using echocardiography criteria. This study included 5926 hospitalized patients referred for echocardiography who had normal or preserved LV systolic function and diastolic function assessment. Of them, 81% had a normal diastolic function or mild dysfunction (grade I), while 19% had advanced LVDD (grade II/III; 13% with grade II and 6% with grade III). It was demonstrated that patients with advanced LVDD were older and more likely to be men and had a higher burden of hypertension, diabetes mellitus, congestive heart failure, atrial fibrillation/flutter and renal failure, abnormal laboratory findings, worse echocardiogram parameters, and longer hospital stay. Furthermore, independent variables associated with advanced LVDD included with increasing age, male sex, significant aortic stenosis, hypertension, and AF/AFL. The present study also shows that the 5-year mortality rate was higher among patients with advanced LVDD vs. normal/LVDD grade I (p_log-rank_ < 0.001). This finding was evident also after adjusting for pertinent variables using the multivariable Cox proportional hazards regression model; LVDD grade II/III was independently associated with a 24% increase in 5-year mortality (HR 1.236, *p* = 0.042). Other variables that were found to be independently associated with increased 5-year mortality were increasing age, renal failure, AF/AFL, and COPD, whereas the male sex and admission to the cardiology ward were associated with a better outcome.

### 4.1. Prevalence of Advanced LVDD

Our study found a relatively low prevalence of LVDD grade III (6%) among the entire cohort. There are several possible explanations for this finding. Firstly, LVDD grade III is generally less common than the less advanced grades. In accordance with our observation, Abubakar et al. studied the prevalence of LVDD among newly diagnosed hypertensive patients in a cross-sectional observational cohort, showing that grade I LVDD was the most common type of LVDD observed, affecting 33% of treatment-naive hypertensive patients; grade II LVDD was less frequent than grade I, affecting 23% of the patients, while grade III LVDD was observed in only 6% of the patients [11]. These findings indicate that even among patients at risk, LVDD is a progressive condition, with advanced grades being less affected. Additionally, as the grading of diastolic dysfunction requires the initial diagnosis of diastolic dysfunction and subsequent testing for the different grades, missing data on the necessary parameters may explain its low prevalence. Also, some of the LVDD grade III patients were excluded from the cohort (especially those with active AF precluding the measurement of A wave), possibly reflecting an underestimation of LVDD grade III. Finally, the measurement of E/A ratio was calculated manually, a process that may be subjected to bias and inconsistencies between different operators, depending on their experience and interpretation skills. Subtle measurement variations might further lead to the under-reporting of cases with higher grades of diastolic dysfunction. Nonetheless, our results of a significantly higher comorbidity burden, as well as unfavorable admission course and outcome, suggest that the grading methods used in the current study are likely valid.

### 4.2. Predictors of Advanced LVDD

Our study revealed that age is one of the significant factors predicting advanced grades of LVDD (OR_adj_ = 1.016 for 1-year increment). Pyszko et al. published a retrospective cohort that studied the effect of age on LVDD in 999 patients, showing that the trans-mitral E/A ratio decreased with age from a mean of 1.65 in subjects aged under 30 years to 0.78 in subjects over 80 years of age (*p* < 0.001) [12]. This demonstrates the well-established observation that diastolic function deteriorates with age presumably due to increased myocardial stiffness and fibrosis. The impairment of diastolic function with increasing age is primarily driven by increased arterial elastance (arterial stiffness), a condition commonly observed in healthy elderly individuals and centenarians. A gradual rise in arterial elastance with age has been described in individuals without known cardiovascular disease. This increased arterial elastance is one of the key pathophysiological mechanisms underlying LV diastolic dysfunction in the elderly [13,14,15].

Similarly, Biernacka et al. showed a strong correlation between aging and advanced LVDD [16]. These findings emphasize the importance of early recognition and potential proactive management among admitted-aged individuals at risk. Other comorbidities that were also independently associated with advanced LVDD include hypertension and AF/AFL. Similar findings were shown by Lin et al. in an observational study demonstrating a higher burden of cardiovascular comorbidities associated with impaired relaxation and filling of the LV [17]. The association between AF/AFL and LVDD is particularly noteworthy, as the loss of atrial contraction during AF rhythm worsens ventricular filling and thus further exacerbates diastolic dysfunction.

### 4.3. Echocardiographic Parameters and Laboratory Work-Up Associated with Advanced LVDD

In addition to the demographics and comorbidity characteristics, the present study identified several echocardiographic indices that differ among patients with varying degrees of LVDD. Patients with advanced LVDD presented with significant changes in LA diameter and volume, elevated E wave and A wave velocities, and E/e′ ratios, as well as lower e′ septal and e′ lateral velocities, variables that comprise the LVDD grading criteria, indicating impaired LV relaxation and elevated filling pressures. Additionally, as expected, significant AS was more prevalent among patients with advanced LVDD and was even identified as an independent predictor of the condition (OR_adj_ = 1.770, *p* < 0.001). These findings corroborate the pathophysiology of LVDD, where progressive stiffening of the LV leads to an increase in LA pressures and subsequent atrial enlargement. The significant difference in the TIG between the subgroups represents pulmonary pressures rising as LVDD worsens, leading to the secondary right heart increased afterload. The finding stresses the role of right-sided strain as part of the pathophysiology of LVDD as right heart systolic dysfunction can potentially complicate management and worsen prognosis. These findings were also noted by several prior studies [18,19,20,21].

Regarding laboratory work-up, we found, as expected, higher NT-pro-BNP levels among admitted patients with advanced LVDD suggesting that these biomarkers may be useful in monitoring disease severity and guiding specific echocardiographic assessment and subsequent treatment decisions. Similarly, it was shown that diastolic dysfunction was predicted by NT-proBNP levels, among other variables [22]. We also showed that patients with advanced LVDD have higher creatinine and potassium levels; these are important findings as chronic kidney disease and the potential subsequent hyperkalemia can limit the use of certain heart failure therapies, including SGLT2 inhibitors, MRAs, and RAAS inhibitors, which may be indicated in patients with LVDD.

### 4.4. Sex Differences and Advanced LVDD

While it is commonly accepted that women have a higher overall prevalence of advanced LVDD [23], some reports showed that diastolic abnormalities were noted to be equally prevalent or more common in males than in females [24,25], in accordance with our study findings. Higher prevalence rates of many shared comorbidities including hypertension, LV hypertrophy, AF/AFL and coronary artery disease in men may partly account for this difference. Nevertheless, in this cohort study, a gender-related association with diastolic abnormalities was confirmed, with significantly higher rates in men than in women. Therefore, gender may be an independent factor in the pathogenesis of diastolic dysfunction, as previously suggested for systolic heart failure and LV hypertrophy [26]. Continued research is necessary to confirm these relationships and assess underlying mechanisms.

### 4.5. Mortality

In accordance with this study’s main hypothesis, 5-year all-cause mortality was significantly higher among patients with advanced LVDD. Our finding is consistent with the natural history of LVDD patients, characterized by heart failure with preserved ejection fraction (HFpEF) with proven progressive unfavorable long-term outcomes [27].

We have also found that after adjusting for pertinent variables, admission to cardiology ward was negatively associated with 5-year mortality (HR 0.466; 95% CI 0.326–0.665, *p* < 0.001). Given that the highest proportion of patients with advanced LVDD were admitted to the cardiology ward (compared to the internal medicine and surgery wards; 23.9% vs. 20.0% vs. 16.2%), it is unlikely that a selection bias played a role in influencing the favorable outcome for these individuals. Similar findings were shown by Bazmpani MA et al. in a prospective observational cohort study including 302 participants admitted with acute decompensated heart failure to internal medicine vs. cardiology ward [28]. The study showed that patients admitted to the internal medicine ward have worse short-term outcomes, including the composite endpoint of in-hospital death and heart failure hospitalizations at 30 days post-discharge in multivariate analysis. The better outcome of patients admitted to the cardiology ward may be attributed to several factors, including access to advanced diagnostic tools, continuous monitoring, and a multidisciplinary approach tailored to the complexities and management of cardiac care. Moreover, specialized cardiac teams are better equipped to initiate evidence-based treatments, such as timely reperfusion strategies in cases of myocardial infarction, which further enhances survival rates. Consequently, early admission to a cardiology ward facilitates effective management of acute cardiovascular issues and improves long-term health outcomes for patients. We found that the year of admission showed no significant effect on mortality in the multivariate Cox regression. This suggests that, from an advanced LVDD perspective, limited evidence-based treatments have been available up until recent years to impact outcomes in this challenging patient population.

Concerning potential evidence-based treatment, mineralocorticoid receptor antagonists (MRA) have been shown to improve unfavorable outcomes among individuals with LVDD [29,30]. Sodium-glucose cotransporter 2 (SGLT2) inhibitors have also shown a similar effect. For example, Lan et al. showed that SGLT2 inhibitors can improve left ventricular (LV) mass index and diastolic function in patients with type 2 diabetes mellitus [31]. However, the effects of renin–angiotensin–aldosterone system (RAAS) inhibitors, such as angiotensin-converting enzyme inhibitors (ACEIs) and angiotensin receptor blockers (ARBs), are still questionable [32]. Some studies have found that LVDD patients benefit from these medications, especially when combined with beta-blockers or calcium channel blockers [33,34], while others showed no significant effect. For example, The VALsartan In Diastolic Dysfunction (VALIDD) randomized controlled trial (RCT) showed no significant difference in the change in diastolic relaxation velocity when valsartan was compared to placebo, concluding that lowering blood pressure improves diastolic function irrespective of the type of antihypertensive agent used [35]. Likewise, the Action in Diabetes and Vascular Disease (ADVANCE) RCT compared the perindopril–indapamide combination to placebo, showing reduced blood pressure and left ventricular mass among patients with diabetes mellitus; however, it showed no improvement in the LV diastolic function [36]. Similarly, the role of angiotensin receptor–neprilysin inhibitors (ARNIs) in treating LVDD remains uncertain [37].

## 5. Limitations

While this study provides significant insights into the prevalence and outcomes associated with LVDD among hospitalized patients, several limitations must be acknowledged. First, the retrospective nature of this study introduces potential biases related to data collection, as the accuracy and completeness of patient records can vary. Furthermore, the reliance on echocardiographic measurements may have limitations, as variations in imaging techniques and interpretation can lead to inconsistencies in the diagnosis and grading of LVDD. Though we did not assess potential variability in the echocardiogram assessment, the differences between the two subgroups suggest that from a clinical perspective, the echocardiogram interpretation was valid. Additionally, this study’s sample is drawn exclusively from hospitalized patients who were referred for an echocardiogram, which may not fully represent the broader population of admitted patients with LVDD. Consequently, the findings may not be generalized to all individuals with diastolic dysfunction, particularly those in community-based settings. Moreover, while this research identified several independent predictors of advanced LVDD, it did not study the potential impact of other confounding factors such as socioeconomic status, lifestyle factors (e.g., diet, physical activity), home medications, and access to healthcare. Also, the reason for admission was not studied. Future prospective studies with more comprehensive data collection and longer follow-up periods are needed to validate these findings and explore the underlying mechanisms driving the observed associations between LVDD and clinical outcomes.

## 6. Conclusions

This study highlights the prevalence and the clinical implications of LVDD among hospitalized patients, revealing associations between advanced LVDD, comorbidities, and an unfavorable admission course. Pertinent variables that were independently associated with advanced LVDD included age, male sex, significant AS, hypertension, and AF/AFL. We have shown that advanced LVDD is a strong predictor for 5-year all-cause mortality after adjusting for pertinent variables. Constituting 19% of the cohort, it is evident that advanced LVDD poses a considerable burden on healthcare systems as well as on patient morbidity and mortality. The findings emphasize the importance of early identification and the potential of comprehensive management strategies for patients with LVDD, particularly those with concomitant risk factors.

Moreover, this study underscores the necessity of echocardiographic evaluations in routine clinical practice among patients at risk, potentially aiding in risk stratification and tailoring management. The increased rates of mortality and hospitalization among patients with advanced LVDD reinforce the urgent need for proactive management, including optimized medical therapy and careful monitoring and control of comorbidities.

Future research should focus on the development of effective strategies for prevention, early intervention and long-term management of LVDD, particularly in high-risk populations.

## Figures and Tables

**Table 1:** Characteristics, comorbidities, hospital course, and long-term follow-up of patients with normal/LVDD grade I vs. advanced LVDD (grades II and III).

Variable	All Patients *n* = 5926	Normal/LVDD Grade I *n* = 4779	LVDD Grade II/III *n* = 1147	*p*-Value
**Patient characteristics**				
Age, years (mean ± SD)	66.0 ± 20.5	64.5 ± 20.1	72.5 ± 21.2	<0.001
Age > 75, *n* (%)	2385 (40.2)	1679 (35.1)	706 (61.6)	<0.001
Male sex, *n* (%)	3229 (54.5)	2498 (52.3)	731 (63.7)	<0.001
**Medical history**				
Hypertension, *n* (%)	2930 (49.4)	2241 (46.9)	689 (51.4)	<0.001
Diabetes mellitus, *n* (%)	1657 (27.9)	1296 (27.1)	361 (31.5)	0.003
Hyperlipidemia, *n* (%)	1431 (24.1)	1150 (23.2)	281 (24.5)	0.248
Smoking, *n* (%)	384 (6.4)	337 (7.1)	47 (4.1)	<0.001
IHD, *n* (%)	830 (14.0)	649 (13.6)	181 (15.8)	0.054
MI, *n* (%)	279 (4.7)	231 (4.8)	48 (4.2)	0.352
CABG, *n* (%)	193 (3.3)	158 (3.3)	35 (3.1)	0.663
PCI, *n* (%)	153 (2.6)	119 (2.5)	34 (3.0)	0.363
CHF, *n* (%)	779 (13.1)	545 (11.4)	234 (20.4)	<0.001
AF/AFL, *n* (%)	713 (12.0)	499 (10.4)	214 (18.7)	<0.001
Stroke/TIA, *n* (%)	1093 (18.4)	904 (18.9)	189 (16.5)	0.056
COPD, *n* (%)	333 (5.6)	267 (5.6)	66 (5.8)	0.825
Renal failure, *n* (%)	1242 (20.9)	963 (20.2)	279 (24.3)	0.002
Obesity, *n* (%)	1026 (17.3)	836 (17.5)	190 (16.6)	0.456
**Hospital course**				
Admission ward				<0.001
Cardiology, *n* (%)	544 (9.2)	414 (8.7)	130 (11.3)	
Internal medicine, *n* (%)	3787 (63.9)	3029 (63.4)	758 (66.1)	
Surgery, *n* (%)	1595 (33.4)	1339 (28.0)	259 (22.6)	
Hospital LOS, days (median [IQR])	8.9 [4.7–16.8]	8.9 [4.6–17.0]	9.1 [5.5–16.8]	0.029
Hospital LOS > 8.9 days, *n* (%)	2965 (50.0)	2389 (50.0)	576 (50.2)	0.889
IV Furosemide, *n* (%)	1881 (31.7)	1491 (30.1)	390 (34.0)	<0.001
**Laboratory work-up**				
NT-proBNP (a), pg/mL (mean ± SD)	433 ± 581	406 ± 567	525 ± 620	0.033
Creatinine (a), mg/dL (mean ± SD)	1.4 ± 1.5	1.4 ± 1.5	1.5 ± 1.7	0.027
Hemoglobin (a), g/dL (mean ± SD)	12.5 ± 2.4	12.6 ± 2.4	12.1 ± 2.3	<0.001
Potassium (a), mEq/L(mean ± SD)	4.1 ± 0.7	4.1 ± 0.6	4.2 ± 0.7	<0.001
Troponin-T (p), ng/L (mean ± SD)	1219 ± 9682	1158 ± 7940	1448 ± 14,507	0.439
TSH (a), mU/L (mean ± SD)	2.7 ± 7.4	2.7 ± 8.0	2.6 ± 3.4	0.365

Abbreviations. a, admission; AF, atrial fibrillation; AFL, atrial flutter; BNP, brain natriuretic peptide; CABG, coronary artery bypass graft; CHF, congestive heart failure; COPD, chronic obstructive pulmonary disease; dL, deciliter; g, gram; IHD, ischemic heart disease; IQR, interquartile range; IV, intravenous; kg, kilogram; L, liter; LOS, length of stay; LVDD, left ventricular diastolic dysfunction; m, meter; mg, milligram; ml, milliliter; MI, myocardial infarction; mEq, milli-equivalent; mU, milliunits; ng, nanogram; p, peak; PCI, percutaneous coronary intervention; pg, picogram; SD, standard deviation; TIA, transient ischemic attack; TSH, thyroid stimulating hormone.

**Table 3:** Unadjusted and adjusted odds ratios with 95% confidence intervals of pertinent baseline characteristics associated with advanced LVDD (grades II and III).

Variable	* Unadjusted		** Adjusted	
	OR_unadj_ (95% CI)	*p*-Value	OR_adj_ (95% CI)	*p*-Value
Age (1-year increment)	1.022 (1.019–1.026)	<0.001	1.016 (1.012–1.020)	<0.001
Male sex	1.605 (1.405–1.833)	<0.001	1.532 (1.337–1.756)	<0.001
Significant AS ***	2.440 (1.872–3.181)	<0.001	1.770 (1.346–2.328)	<0.001
AF/AFL	1.967 (1.651–2.344)	<0.001	1.541 (1.285–1.849)	<0.001
HTN	1.704 (1.494–1.942)	<0.001	1.224 (1.054–1.421)	0.008
DM	1.234 (1.073–1.420)	0.003		
Renal failure	1.274 (1.094–1.483)	0.002		
Hyperlipidemia	1.091 (0.941–1.266)	0.248		
IHD	1.192 (0.997–1.426)	0.054		
Obesity	0.936 (0.788–1.113)	0.456		
COPD	1.032 (0.782–1.362)	0.825		
Stroke/TIA	0.846 (0.712–1.004)	0.056		

* Unadjusted odds ratios (OR) with 95% confidence interval (CI of variables associated with advanced LVDD. ** Using multivariate logistic regression analysis (OR and 95% CI) to identify variables independently associated with advanced LVDD including. The model included variables that were found significant in the unadjusted OR_unadj_ (*p* ≤ 0.05) using the enter method. *** Defined as moderate-severe or severe aortic stenosis. Abbreviations as in Table 1 and OR_unadj_, unadjusted odds ratio; OR_adj_, adjusted odds ratio.

## Data Availability

The data presented in this study are available on request from the corresponding author.

## Supplementary Materials

The following supporting information can be downloaded at https://www.mdpi.com/article/10.3390/jcm14041096/s1, Figure S1: Pertinent demographic variables and comorbidities associated with advanced LVDD. The figure presents unadjusted odds ratios (ORs) with 95% confidence interval (CI); Figure S2: Independent variables associated with advanced LVDD; Figure S3: Prevalence of 5-year mortality and survival across pertinent demographics and comorbidities; Figure S4: Unadjusted hazard ratios (HRs) with 95% confidence interval (CI) of pertinent variables including advanced LVDD associated with 5-year mortality; Table S1: Characteristics, comorbidities, hospital course and long-term follow-up of patients with normal/LVDD grade I vs. advanced LVDD (grades II and III) for those who met the primary outcome or completed the 5-year follow-up; Table S2. Echocardiographic parameters of patients with normal/LVDD grade I vs. advanced LVDD (grade II and III) for those who met the primary outcome or completed the 5-year follow-up; Table S3: Association of pertinent variables with 5-year survival and mortality: Crude and univariate Cox proportional hazards model calculating HR with 95% CI; Table S4: Independent predictors associated with 5-year mortality.
