# Supplementary material for: Prevalence and Long-Term Prognostic Significance of Advanced Diastolic Dysfunction Among Hospitalized Patients Referred for Echocardiography
Source: J Clin Med. 2025 Feb 8;14(4):1096. doi: 10.3390/jcm14041096 (PMC11856506; doi:10.3390/jcm14041096)
Supplement: Supplementary file 1 [file jcm-14-01096-s001.zip › jcm-3426706-supplementary.pdf]

Prevalence and long-term prognostic significance of advanced diastolic dysfunction among hospitalized patients referred for echocardiography- Supplementary data

Supplementary Table S1. Characteristics, comorbidities, hospital course and long-term follow-up of patients with normal/LVDD grade I vs advanced LVDD (grades II and III) for those who met the primary outcome or completed the 5-year follow-up

Variable	All patients n=2,745	Normal/LVDD grade I n=2,206	LVDD grade II/III n=539	*p*-value
**Patient characteristics**				
Age, years (mean ± SD)	67.7±20.8	66.0±20.4	74.5±20.9	**<0.001**
Age >75, n (%)	1,186 (43.2)	840 (38.1)	346 (64.2)	**<0.001**
Male sex, n (%)	1,496 (54.5)	1,159 (52.5)	337 (62.5)	**<0.001**
**Medical history**				
Hypertension, n (%)	1,387 (50.5)	1,047 (47.5)	340 (63.1)	**<0.001**
Diabetes mellitus, n (%)	810 (29.5)	637 (28.9)	173 (32.1)	0.142
Hyperlipidemia, n (%)	703 (25.6)	570 (25.8)	133 (24.7)	0.579
Smoking, n (%)	188 (6.8)	162 (7.3)	36 (4.8)	**0.038**
IHD, n (%)	421 (15.3)	319 (14.5)	102 (18.9)	**0.010**
MI, n (%)	135 (4.9)	114 (5.2)	21 (3.9)	0.221
CABG, n (%)	97 (3.5)	75 (3.4)	22 (4.1)	0.442
PCI, n (%)	81 (3.0)	58 (2.6)	23 (4.3)	**0.044**
CHF, n (%)	464 (16.9)	326 (14.8)	138 (25.6)	**<0.001**
AF/AFL, n (%)	369 (13.4)	257 (11.7)	112 (20.8)	**<0.001**
Stroke/TIA, n (%)	442 (16.1)	364 (16.5)	78 (14.5)	0.251
COPD, n (%)	196 (7.1)	158 (7.2)	38 (7.1)	0.928

Renal failure, n (%)	647 (23.6)	498 (22.6)	149 (27.6)	**0.013**
Obesity, n (%)	390 (14.2)	315 (14.3)	75 (13.9)	0.743
**Hospital course**				
Admission ward				**0.001**
Cardiology, n (%)	175 (6.4)	130 (5.9)	45 (8.4)	
Internal medicine, n (%)	1,871 (68.2)	1,485 (67.3)	386 (71.6)	
Surgery, n (%)	699 (25.4)	591 (26.8)	108 (20.0)	
Hospital LOS, days (median [IQR])	8.9 [4.8-16.8]	7.9 [3.9-15.0]	12.1 [6.7-22.4]	0.311
Hospital LOS>8.9 days, n (%)	1,477 (53.8)	1,176 (53.3%)	301 (55.8)	0.290
IV Furosemide, n (%)	809 (29.5)	610 (27.7)	199 (36.9)	**<0.001**
**Laboratory work-up**				
NT-proBNP (a), pg/ml (mean ± SD)	471±570	426±526	611±674	**0.009**
Creatinine (a), mg/dl (mean ± SD)	1.5±1.6	1.5±1.6	1.6±1.8	0.080
Hemoglobin (a), g/dl (mean ± SD)	12.2±2.4	12.3±2.5	11.8±2.3	**<0.001**
Potassium (a), mEq/L(mean ± SD)	4.2±0.7	4.1±0.6	4.3±0.7	**<0.001**
Troponin-T (p), ng/L (mean ± SD)	868±5224	834±5359	998±5676	0.578
TSH (a), mU/L (mean ± SD)	3.0±9.4	3.1±10.4	2.7±3.6	0.516

Abbreviations. a, admission; AF, atrial fibrillation; AFL, atrial flutter; BNP, brain natriuretic peptide; CABG, coronary artery bypass graft; CHF, congestive heart failure; COPD, chronic obstructive pulmonary disease; dl, deciliter; g, gram; IHD, ischemic heart disease; IQR, interquartile range; IV, intravenous; kg, kilogram; L, liter; LOS, length of stay; LVDD, left ventricular diastolic dysfunction; m, meter; mg, milligram; ml, milliliter; MI, myocardial infarction; mEq, milli-equivalent; mU, milliunits; ng, nanogram; p, peak; PCI, percutaneous coronary intervention; pg, picogram; SD, standard deviation; TIA, transient ischemic attack; TSH, thyroid stimulating hormone.

Supplementary Table S2. Echocardiographic parameters of patients with normal/LVDD grade I vs advanced LVDD (grade II and III) for those who met the primary outcome or completed the 5-year follow-up

Variable	All patients n=2,745	Normal/LVDD grade I n=2,206	LVDD grade II/III n=539	*p*-value
HR, bpm (mean ± SD)	76.0±16.6	76.6±16.9	73.6±15.0	**0.008**
Significant AS, %*	151 (5.5)	103 (4.7)	48 (8.9)	**<0.001**
**Ventricular characteristics**				
LVDD, cm (mean ± SD)	4.6±1.8	4.7±2.0	4.5±0.6	**0.037**
LVSD, cm (mean ± SD)	2.8±0.5	2.9±0.5	2.8±0.6	**0.047**
LVPWD, cm (mean ± SD)	1.0±0.2	1.0±0.2	1.0±0.2	**0.044**
IVSD, cm (mean ± SD)	1.2±0.2	1.2±0.2	1.3±0.2	**<0.001**
LVEF, % (mean ± SD)	58.0±4.0	58.2±3.9	57.5±4.3	**<0.001**
**Atrial characteristics**				
LA diameter, cm (mean ± SD)	4.0±0.9	3.9±0.6	4.2±1.5	**<0.001**
LA area, cm2 (mean ± SD)	23.9±8.6	22.7±6.9	25.6±10.7	0.215
LA volume, cm3 (mean ± SD)	72.7±49.5	69.8±37.9	78.7±67.4	0.059
LA volume index, ml/m2 (mean ± SD)	40.0±34.5	37.5±20.6	45.0±52.0	**0.027**
LA volume index>34 ml/m2, %	9.1	6.6	19.5	**<0.001**
**Waves**				
E wave velocity, cm/s (mean ± SD)	85.0±96.7	82.1±26.4	96.8±211.5	**0.002**
A wave velocity, cm/s (mean ± SD)	83.9±26.3	82.0±22.4	91.5±37.4	**<0.001**
e′ septum velocity, cm/s (mean ± SD)	6.9±2.4	7.1±2.4	6.3±2.6	**<0.001**
e′ lateral velocity, cm/s (mean ± SD)	8.9±3.5	9.1±3.4	8.0±3.6	**<0.001**
**Waves ratio**				

E/A ratio (mean ± SD)	1.1±1.6	1.0±0.4	1.5±3.6	**<0.001**
E/A ratio>2, %	5.9	0	29.9	**<0.001**
E/e′ ratio (mean ± SD)	11.6±5.7	11.1±5.5	13.5±6.2	**<0.001**
E/e′ ratio>14, %	22.6	19.4	35.8	**<0.001**
**Gradient**				
TIG, mmHg (mean ± SD)	35.0±15.5	33.2±15.2	41.4±15.2	**<0.001**
TIG>28 mmHg, %	59.3	52.5	83.7	**<0.001**

* Defined as moderate-severe or severe aortic stenosis

Abbreviations. bpm, beat per minute; cm, centimeter; HR, heart rate; IVSD, interventricular septal diameter; LA, left atrium, LVDD, left ventricular diastolic dysfunction; LVEF, left ventricular ejection fraction, LVDD, left ventricular diastolic diameter; LVSD, left ventricular systolic diameter; LVOT, left ventricular outflow tract; LVPWD, left ventricular posterior wall diameter; m, meter; ml, millimeter; mmHg, millimeter of mercury; SA, short axis; SD, standard deviation; TIG, tricuspid insufficiency gradient.

Supplementary Table S3. Association of pertinent variables with 5-year survival and mortality: Crude and univariate Cox proportional hazards model calculating HR with 95% CI

Variable	**All n=5,926**	**5-year survival n=4,679**	**5-year mortality n=1,247**	Unadjusted HR*	95% CI	*p*-value
**Advanced LVDD, n (%)**	1147 (19.4)	853 (18.2)	294 (23.6)	1.392	1.221-1.586	**<0.001**
Age, years (mean ± SD)	66.0±20.5	63.6±20.6	75.2±17.7	1.033	1.033-1.037	**<0.001**
Male sex, n (%)	3,229 (54.5)	2,562 (54.8)	667 (53.5)	0.953	0.852-1.065	0.425
DM, n (%)	1,657 (28.0)	1,216 (26.0)	441 (35.4)	1.471	1.310-1.652	**<0.001**
HTN, n (%)	2,930 (49.4)	2,197 (47.0)	733 (58.8)	1.528	1.365-1.711	**<0.001**
Hyperlipidemia, n (%)	1,431 (24.1)	1,080 (23.1)	351 (28.1)	1.249	1.104-1.413	**<0.001**
AF/AFL, n (%)	713 (12.0)	494 (10.6)	219 (17.6)	1.740	1.504-2.014	**<0.001**
IHD, n (%)	830 (14.0)	631 (13.5)	199 (16.0)	1.176	1.010-1.368	**0.025**
CABG, n (%)	193 (3.3)	165 (3.5)	28 (2.2)	0.614	0.422-0.893	**0.024**
Renal failure, n (%)	1,242 (21.0)	832 (17.6)	410 (32.9)	2.087	1.854-2.348	**<0.001**
COPD, n (%)	333 (5.6)	214 (4.6)	119 (9.5)	1.936	1.602-2.338	**<0.001**
Stroke/TIA, n (%)	1,093 (18.4)	892 (19.1)	201 (16.1)	0.836	0.719-0.972	**0.017**
Obesity, n (%)	1,026 (17.3)	848 (18.1)	178 (14.3)	0.773	0.628-0.952	**0.015**
Cardiology ward admission, n (%)	544 (9.2)	482 (10.3)	62 (5.0)	0.526	0.407-0.679	**<0.001**

* Univariate Cox proportional-hazards model was calculated for each pertinent variable to assess the HR and 95% CI for up to 5-year mortality.

Abbreviations. AF, atrial fibrillation; AFL, atrial flutter; BMI, body mass index; CABG, coronary artery bypass grafting; CI, confidence interval; DM, diabetes mellitus; HR, hazard ratio; HTN, hypertension; IHD, ischemic heart disease; LVDD, left ventricular diastolic dysfunction; n, number; TIA, transient ischemic attack.

Supplementary Table S4. Independent predictors associated with 5-year mortality*

Variable	Adjusted HR	95% CI	*p*-value
**Advanced LVDD**	1.236	1.008-1.517	**0.042**
Renal failure	1.763	1.422-2.157	**<0.001**
COPD	1.573	1.151-2.148	**0.004**
AF/AFL	1.358	1.072-1.719	**0.011**
Age (1-year increment)	1.036	1.029-1.044	**<0.001**
Male sex	0.771	0.633-0.938	**0.012**
Cardiology ward admission	0.466	0.326-0.665	**<0.001**
Stroke/TIA	0.784	0.609-1.009	0.059
Hypertension	0.896	0.720-1.117	0.329
Diabetes mellitus	1.134	0.920-1.397	0.240
Hyperlipidemia	1.008	0.813-1.250	0.942
Obesity	0.866	0.696-1.078	0.198
Ischemic heart disease	1.089	0.822-1.443	0.551
CABG	0.579	0.316-1.060	0.077

* Multivariate Cox proportional-hazards regression model with HR and 95% CI was used to calculate the time to all-cause mortality or up to 5 years, whichever came first. Variables included in the model were: sex and variables found significant ($p \leq 0.05$) in the univariate HR analysis. To account for potential year and time-related biases, the year of admission was included in the multivariate analysis as a covariate.

Abbreviations as in **Supp. Table S1**.

Supplementary Figure S1. Pertinent demographic variables and comorbidities associated with advanced LVDD. The figure presents the unadjusted odds ratios (OR) with 95% confidence interval (CI).

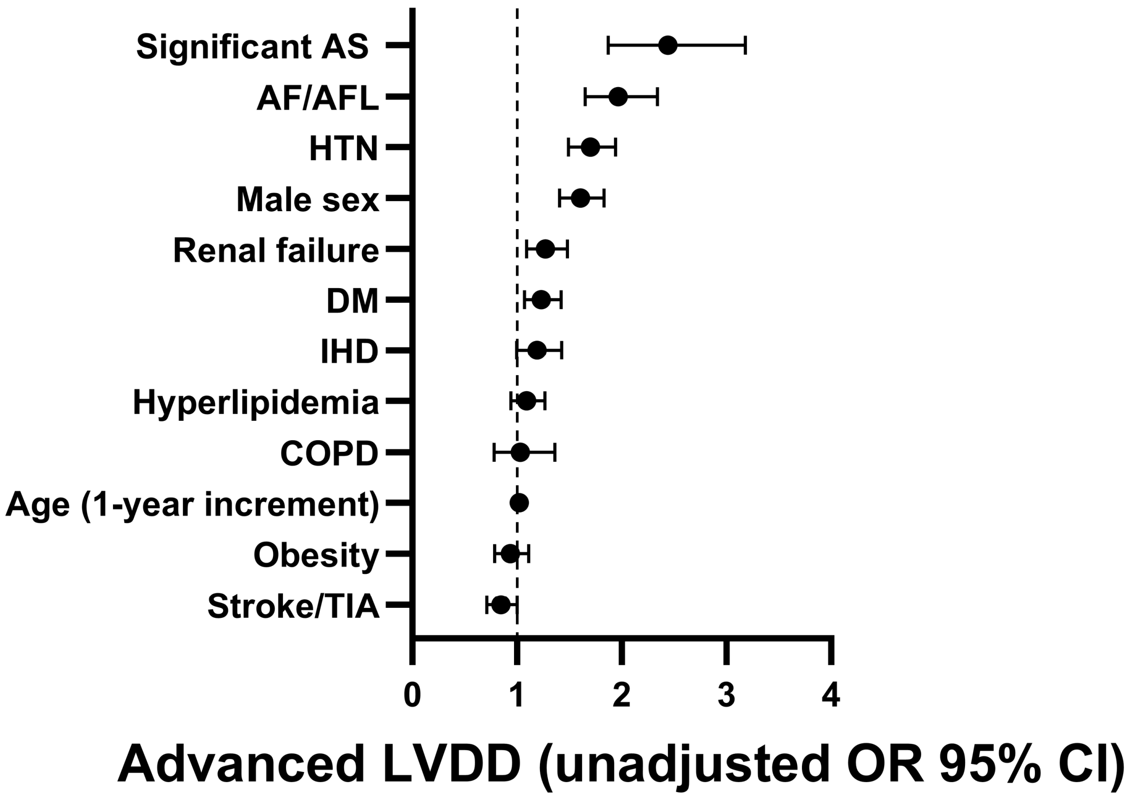

Abbreviations as in **Supp. Table S1**.

Supplementary Figure S2. Independent variables associated with advanced LVDD*

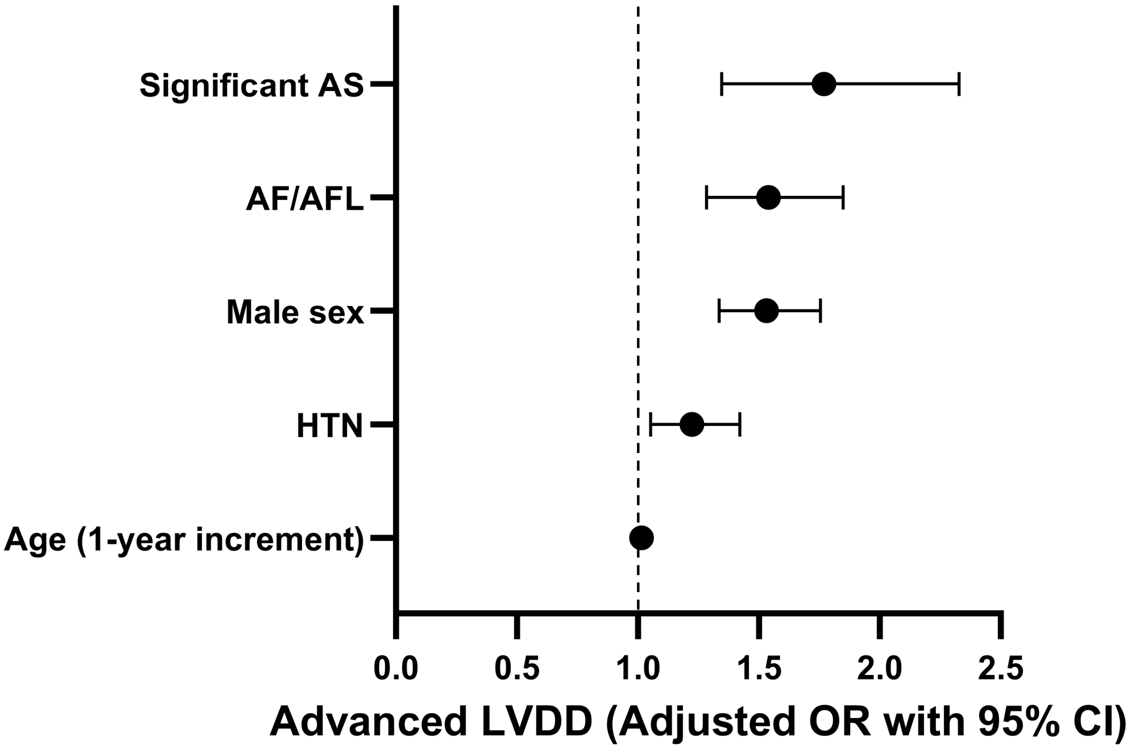

* Multivariate logistic regression analysis (OR and 95% CI) was calculated to test the association of identified variables independently associated with advanced LVDD (p ≤0.05) using the enter method (See **Materials and Methods** and **Table 3**).

Abbreviations as in **Supp. Table S1**.

Supplementary Figure S3. Prevalence of 5-year mortality and survival across pertinent demographics and comorbidities

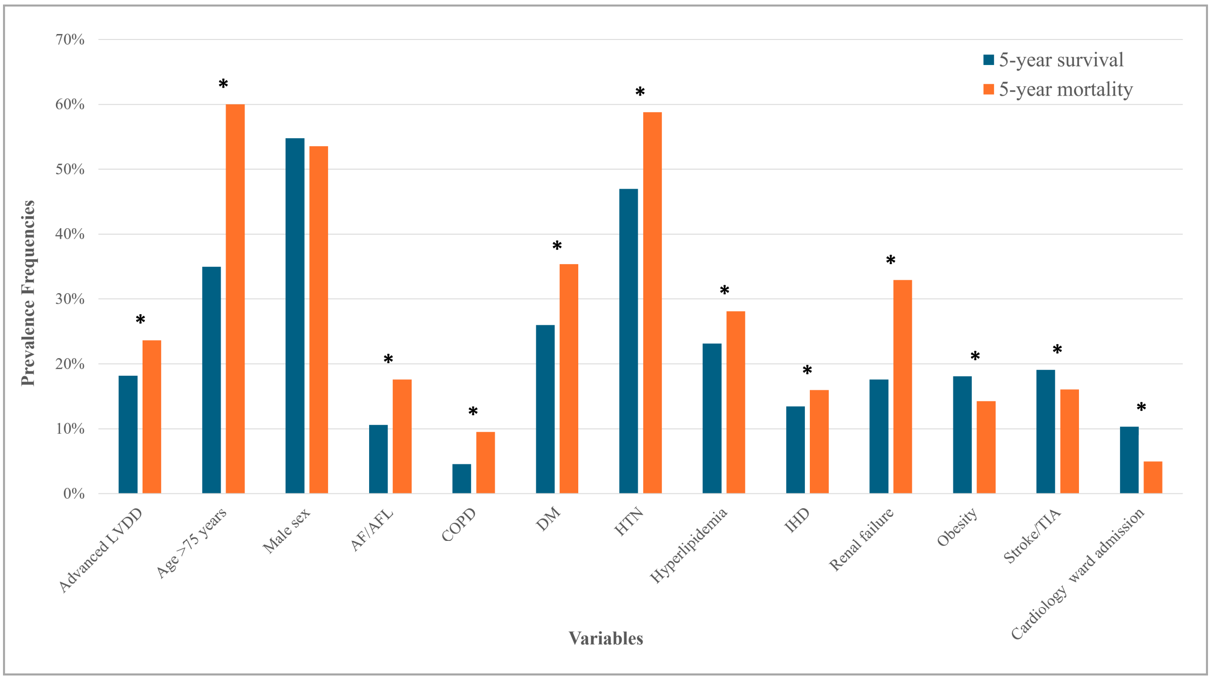

* Comparisons with a significant p-value (≤0.05).

Abbreviations as in **Supp. Table S1**.

Supplementary Figure S4. Unadjusted hazard ratios (HR) with 95% confidence interval (CI) of pertinent variables including advanced LVDD associated with 5-year mortality*

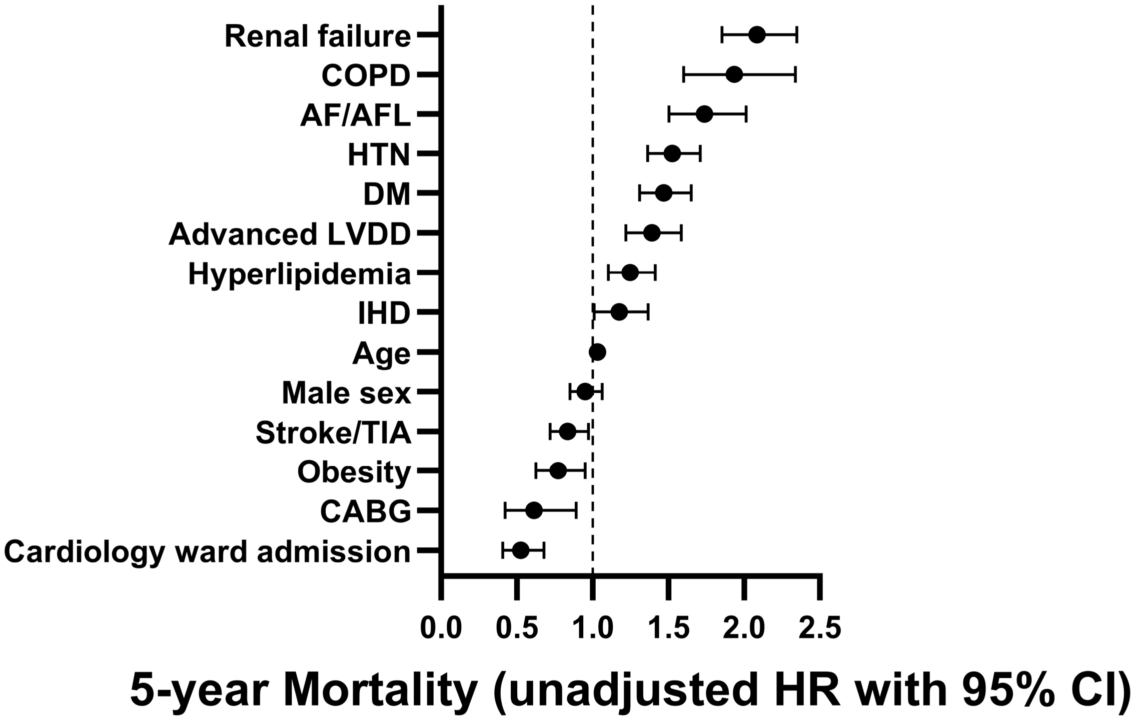

* A univariate Cox proportional-hazards model was calculated for each of the pertinent variables to assess the HR and 95% CI for 5-year mortality.

Abbreviations as in **Supp. Table S1**
